# ArrayMining: a modular web-application for microarray analysis combining ensemble and consensus methods with cross-study normalization

**DOI:** 10.1186/1471-2105-10-358

**Published:** 2009-10-28

**Authors:** Enrico Glaab, Jonathan M Garibaldi, Natalio Krasnogor

**Affiliations:** 1 School of Computer Science, Nottingham University, Jubilee Campus, Wollaton Road, Nottingham, UK

## Abstract

**Background:**

Statistical analysis of DNA microarray data provides a valuable diagnostic tool for the investigation of genetic components of diseases. To take advantage of the multitude of available data sets and analysis methods, it is desirable to combine both different algorithms and data from different studies. Applying ensemble learning, consensus clustering and cross-study normalization methods for this purpose in an almost fully automated process and linking different analysis modules together under a single interface would simplify many microarray analysis tasks.

**Results:**

We present ArrayMining.net, a web-application for microarray analysis that provides easy access to a wide choice of feature selection, clustering, prediction, gene set analysis and cross-study normalization methods. In contrast to other microarray-related web-tools, multiple algorithms and data sets for an analysis task can be combined using ensemble feature selection, ensemble prediction, consensus clustering and cross-platform data integration. By interlinking different analysis tools in a modular fashion, new exploratory routes become available, e.g. ensemble sample classification using features obtained from a gene set analysis and data from multiple studies. The analysis is further simplified by automatic parameter selection mechanisms and linkage to web tools and databases for functional annotation and literature mining.

**Conclusion:**

ArrayMining.net is a free web-application for microarray analysis combining a broad choice of algorithms based on ensemble and consensus methods, using automatic parameter selection and integration with annotation databases.

## Background

DNA microarray experiments provide a powerful means to improve our understanding of diseases with a genetic basis or contribution. Commercial microarray chips for highly accurate diagnosis of several cancers are already available on the market [1,2] and pharmaceutical companies are using DNA-chip technology to identify new drug targets.

The fast accumulation of gene expression data in public online databases and the great variety of available analysis methods, however, also pose new challenges. Integrating data from different sources, choosing appropriate normalization, analysis and cross-validation methods and selecting suitable parameters requires substantial time and effort. Since different algorithms have different strengths and similar data from independent studies is often available, it is desirable to combine multiple methods and/or data sets to obtain more robust and accurate results. This creates ample opportunities for ensemble methods and cross-study normalization techniques.

Although statistical programming frameworks like R [3] and Matlab [4] allow users to develop and apply complex scripts for expression data analysis, they are difficult to use for non-experts and there is a high risk of deviating from standard guidelines. To obviate the need for specialized programming skills and manual software installations, several web-based tools for gene expression analysis have been presented in recent years. Currently available integrative online analysis services include *GEPAS *[5], *Expression Profiler *[6], *ASTERIAS *[7], *EzArray *[8], *CARMAweb *[9], *MAGMA *[10], *ArrayPipe *[11], *RACE *[12], *WebArray *[13] and *MIDAW *[14]. These web-based systems provide methods for a multitude of data pre-processing and analysis purposes ranging from image analysis, missing value imputation, single-study normalization, gene filtering and gene name conversion to higher-level analysis methods for clustering, gene selection and gene annotation, prediction, data visualization and gene set enrichment analysis, among others.

Additionally, numerous web-applications have been developed and optimized for single, specific analysis tasks, e.g. biclustering of genes and samples [15], co-clustering of genes with similar functional annotation [16], framework inference for regulatory networks [17] and cross-species clustering [18]. Although various tools provide a choice and comparison between different algorithms for one analysis task, to the best of our knowledge, currently no integrative analysis software enables the user to easily combine multiple methods together using ensemble learning and consensus clustering techniques. Previous studies have shown that microarray analysis can profit from ensemble feature selection, ensemble prediction and consensus clustering methods both in terms of robustness and accuracy [19-22], suggesting that there is significant potential still to be exploited with these approaches.

Similarly, it would be desirable not only to combine different algorithms but also different data sets for a common organism and phenotype. Although currently available cross-study normalization methods are based on simplified assumptions and limited in applicability and accuracy, various successful applications [23,24] have shown that the benefits of an increased sample size can outweigh the loss of information due to the normalization process.

For these reasons, we have developed a new web-application that provides access to multiple algorithms for each of the most common tasks in statistical microarray analysis, namely gene selection, sample clustering, sample classification and gene set analysis, based on a single, easy-to-use interface. In contrast to other web-tools, in which the results of individual methods are made available, here, ensemble feature selection, ensemble prediction and consensus clustering approaches are provided. Likewise, instead of using only data from a single study, different cross-study normalization methods are made available to integrate similar data from different studies and compare the results based on density and quantile-quantile plots.

Apart from these combinations of data sets and methods within an analysis module, different modules have been interlinked, enabling for example the integration of gene set analysis with classification or cross-study analysis with gene selection or clustering. Other new features include access to an in-house developed rule-based evolutionary classification algorithm, automatic parameter selection mechanisms on all modules, the availability of specific cancer-related gene sets for enrichment analysis in addition to gene sets from KEGG and GO, and a 3D-VRML-visualization of clustering results using the authors' new R software package "vrmlgen" [25].

Since the above methods and features are not available on other microarray-related web-tools, and similarly, other tool sets include methods distinct from our system, we see our service as a complement rather than an alternative to existing services.

In the following we provide an overview of the workflow and describe all features in detail.

## Implementation and workflow

The ArrayMining.net tool set consists of five main modules for microarray analysis: *Cross-Study Normalization*, *Gene selection*, *Class Discovery*, *Class Assignment *and *Gene Set Analysis*. Each of these modules features multiple analysis methods accessible through a unified web-interface. The user can upload his own data in tab-delimited text-file format or as zip-compressed Affymetrix CEL-files which will be automatically extracted, normalized and summarized using the Robust Microarray Analysis (RMA) method [26]. Alternatively, various example data sets have been made available directly on the webpage and access to the GEO database [27], the largest public microarray data base, is provided on the class discovery module. After submitting an analysis task, an output webpage containing the downloadable results as plots, tables, VRML-files etc. is generated. Depending on the chosen module and algorithm the data can be forwarded to further analysis modules and will be interlinked with annotation data from external web-tools and data bases.

ArrayMining.net is based on software written in the programming languages R [3] and C++ and a PHP-interface combining all implementations together on an Apache web server. The system uses in-house algorithms and implementations as well as standard packages from the Bioconductor project [28]. All modules are easily extensible and the authors encourage users to contribute with feature requests or their own analysis scripts. A regularly updated illustration of the workflow and features on our server is available online (see Availability section). Below we describe each of the modules in detail.

### Cross-study normalization module

Current microarray studies often only contain a small number of samples, resulting in limited robustness and reliability of statistical analyses. To alleviate this problem five cross-study normalization methods have been made available on ArrayMining.net to combine samples from two different studies: An approach based on linked gene- and sample-clustering (XPN [23]), an empirical Bayes method (EB [29]), a median rank score based method (MRANK [24]), an outlier-removing discretization technique (NorDi [30]) and a quantile discretization procedure (QDISC [24]). While the first three methods provide continuous-valued outputs, the last two are based on discretization to filter out noise, exploiting the fact that for higher-level analysis often only a general categorization of gene expression levels in different conditions is required (e.g. "unaltered", "up"- or "down"-regulated), but potentially resulting in a higher loss of biological information. The input data sets can originate from different microarray platforms, but the associated gene sets need to overlap significantly and the samples should be derived from the same tissue type under comparable biological conditions. As a result, the combined data can be downloaded or forwarded to other modules, and density and quantile-quantile plots are generated to compare different algorithms.

### Gene selection module

**Figure 1:**
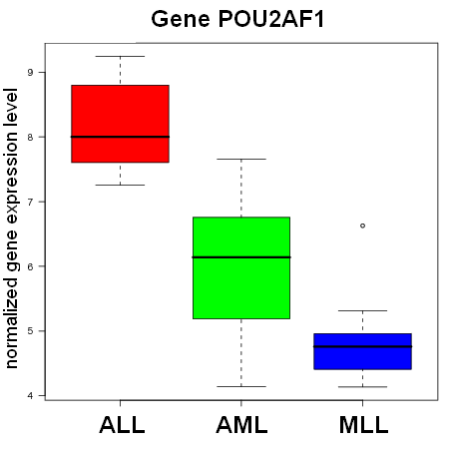
**Boxplots**. Example of a boxplot illustrating the spread of a gene's expression values across three classes of leukemia samples: Acute Lymphoblastic Leukemia (ALL), Acute Myeloid Leukemia (AML) and Mixed Lineage Leukemia (MLL) (data set by Armstrong et al. [55], [see Additional file 1 for further details on this and other differentially expressed genes]).

**Figure 2:**
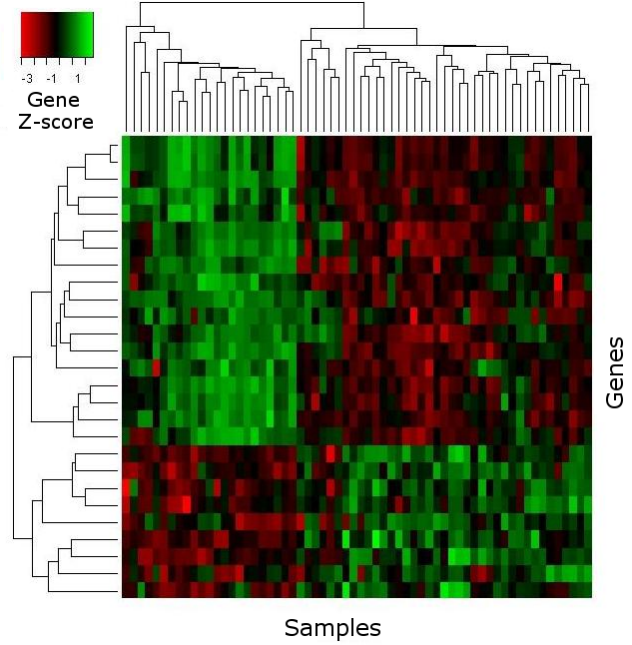
**Heat map**. Example of a heat map visualizing the expression values of selected genes (rows) across samples (columns).

Identifying differentially expressed genes is a common starting point for the biological interpretation of microarray data. Our gene selection module enables the comparison and combination of a diverse choice of methods for this purpose: The Empirical Bayes t-statistic (*eBayes*) [31,32], the Significance Analysis in Microarrays method (*SAM*) [33], a correlation-based combinatorial feature selection approach (*CFS*) [34], a ranking method based on Random Forest classification (*RF-MDA*) [35] and a Partial-Least-Squares based filter (*PLS-CV*) [36] using the weight vectors defining the first latent components in cross-validated PLS-models. To exploit the synergies of different algorithms, we have implemented a method to compute aggregated gene ranks from the sum of ranks of individual methods (*ENSEMBLE*). The resulting outcome reports provide a ranked list of genes, in which known gene identifiers become clickable navigation items, referring the user to related entries in functional annotation databases and literature search engines. Additionally, box plots and heat maps (see Fig. 1 and 2) visualize the expression values of top-ranked genes across different sample-groups. If the supplied data uses common gene identifiers (Entrez Gene ID, NCBI GI accession, Unigene ID, RefSeq Genomic ID, etc.), the list of selected genes can be forwarded to external analysis tools, e.g. the functional annotation clustering service of the DAVID web database [37].

### Class discovery module

**Figure 3:**
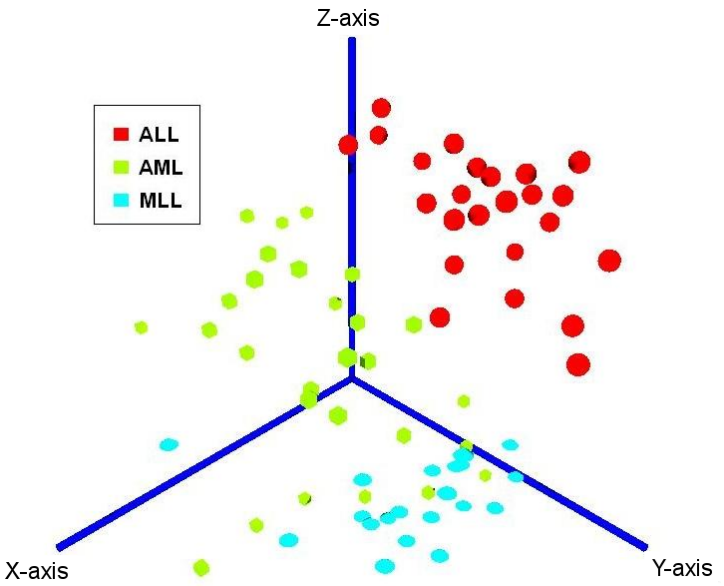
**Independent Component Analysis**. Example of a VRML-visualization for an Independent Component Analysis (data set by Armstrong et al. [55]).

Clustering methods allow experimenters to identify natural groupings among microarray samples based on their expression patterns across the genes. To account for the great variety of existing scoring and search space exploration methods, our class discovery module includes both partition-based and hierarchical clustering algorithms, an evaluation based on multiple validity indices and a consensus clustering method. Currently, the partition-based clustering methods available are *k-Means*, *PAM *[38], *SOM *[39] and *SOTA *[40], and the hierarchical clustering methods are *Average Linkage Agglomerative Clustering*, *Divisive Analysis Clustering *and a combination between the agglomerative and divisive approach, *Hybrid Hierarchical Clustering *[41]. To combine the information content from multiple clusterings into a single representative solution, we have implemented our own consensus clustering approach, which maximizes a score for the agreement between sample-pair assignments of the consensus clustering and all input clusterings using a fast simulated annealing approach [42]. This method was developed based on experiences from earlier work on protein structure similarity clustering [43], which showed that consensus methods can increase the robustness and reliability of statistical analyses on biological data sets. For each algorithm the number of clusters is estimated automatically by means of multiple validity indices and a refined estimate can be obtained by combining all pairs of algorithms and validity indices. Optionally, different types of data standardization and two gene filtering methods can be applied prior to the analysis. This includes a classical variance-based filter as well as a recently published parameter-free method, which can distinguish between uncorrelated, uninformative genes and regulators with high correlation to other genes [44]. An alternative filtering approach is to first use the gene set analysis module (see below) to extract "meta-genes" representing biological pathways and forward this data to the class discovery module. As a result for each analysis, the user will obtain a tabular summary of the calculated validity indices and clustering results and various graphical outputs including a silhouette-plot [45], a 2D principal components plot and a 3D VRML-visualization (see Fig. 3), including density estimation contour surfaces based on an Independent Component Analysis of the data and our software-package "vrmlgen" for the R programming language [3] (freely available at Ref. [25] and the official R package archive, CRAN).

### Class Assignment module

An important goal behind microarray analysis is to improve the diagnosis of diseases with genetic components by predicting the disease type based on labeled training data. The third module on our web-server is therefore dedicated to supervised learning methods, including various common methods for microarray sample classification (*SVM *[46], *RF *[35], *PAM *[38] and *kNN*). We also provide access to an in-house developed rule-based machine learning approach, BioHEL [47], which learns structured classification rule sets, known as "decision lists", by applying a genetic algorithm within an iterative rule learning (IRL) framework. BioHEL has previously been shown to achieve high prediction accuracies on complex biological data sets [48], while being based on easily interpretable "if-then-else"-rules. The prediction methods can be evaluated and compared based on the widely accepted external two-level cross-validation methodology [49], using automatic parameter optimization within a nested cross-validation. As with the other modules, an ensemble of algorithms is available both for selection and prediction to obtain more robust results. Moreover, since prediction models derived from training data of a single study can typically not be applied to samples from other platforms and laboratories, the combination of cross-study normalization (see above) with prediction provides a means to obtain more general models based on a larger sample size.

The results for an analysis contain various performance measures for evaluation and Z-scores for the genes that were most frequently selected across different cross-validation cycles. To obtain more insights on these genes, similar analysis plots and annotation tools are available as for the gene selection module.

### Gene Set Analysis module

Two common problems in microarray analysis are high noise levels for single genes and a high number of redundant or uninformative genes. Using gene set analysis (GSA) to aggregate functionally related genes into gene sets and summarizing their expression values to a robust "meta"-gene expression vector is a promising approach to overcome some of these limitations [50]. Moreover, differentially expressed gene sets can provide insights on the differences between the biological conditions of the samples on the level of molecular modules and biochemical pathways. Our gene set analysis module provides access to three functional annotation sources to identify functionally related genes in a data set and extract corresponding gene sets: The Gene Ontology Database [51], the KEGG data base [52], and a collection of 37 cancer-related gene sets from the van Andel Institute in Michigan [53]. Alternatively, users can specify their own gene sets using the gene identifiers for the data set of interest. Since common non-parametric GSA methods are often computationally expensive or provide only rough estimates of a gene's significance score, we compute p-values based on the parametric PAGE-method [53], requiring a minimum gene set size of approx. 10 genes. To adjust for multiple testing, the Benjamini-Hochberg method [54] is used.

Summarized meta-gene expression vectors for a gene set are obtained by transforming the expression levels using Principal Component Analysis (PC-GSA) or Multidimensional Scaling (MDS-GSA).

The outcome is presented as a ranked list of gene sets and additionally contains box plots and heat maps similar to those on the gene selection module. Meta-gene expression values derived from the gene sets can be downloaded or forwarded to other analysis modules, e.g. to be used as predictors in sample classification.

## Results

Providing example results for all modules and algorithms on ArrayMining.net would exceed the scope of this paper. However, we have included some results obtained with the well-known three-class leukemia data set by Armstrong et al. [55] [see Additional file 1]. This includes the list of the 30 top-ranked genes using the ENSEMBLE gene selection method, as well as a heat map, box plots, a ranked list of cancer gene sets from the gene set analysis module, sample classification results, a VRML-file visualizing the results from an Independent Component Analysis computed on the class discovery module (shown in Fig. 3) and a discussion of all results. In summary, nearly all of the selected genes with available annotation data are known or likely to be differentially expressed in different leukemia types. An example box plot for a top-ranked gene - the transcriptional regulator POU2AF1, which has been implicated in lymphoma and leukemia development [56] - is shown in Fig. 1. We also show results for a combination of two modules, obtaining an average sample classification accuracy of 87% (external 10-fold cross-validation) on the Class Assignment module when using meta-genes derived from the gene set analysis module as robust input features. On the class discovery module, the clustering and validity methods were able to perfectly distinguish two leukemia subtypes in the data, Mixed Lineage Leukemia (MLL) and Acute Lymphoblastic Leukemia (ALL), while the samples for a third subtype, Acute Myeloid Leukemia (AML), could only partly be separated from the other two groups. However, when visualizing the pre- filtered data using an Independent Component Analysis, the three leukemia groups were well separated in 3D-space with only a small overlap between the MLL and the AML group, although all results were generated in a fully automatic process [see Additional file 2 for a VRML-visualization of the data].

Since these examples cover only some of the available features, various well-known microarray cancer data sets are available on the different analysis modules to enable the user to more fully explore the capabilities of ArrayMining.net without needing to upload new data.

## Conclusion

We have developed a new web-application that provides a simple and fast way to analyze arbitrary DNA-chip data and other high-dimensional data sets. Ensemble, consensus and cross-study normalization methods help to increase the robustness and accuracy of the outcomes, and automatic parameter selection mechanisms and a direct linkage to functional annotation data bases (ENSEMBL, DAVID, etc.) relieve the user of time-consuming routine tasks. For each of the major statistical analysis tasks - feature selection, clustering, prediction and gene set analysis - several analysis methods are available and can be compared, combined or interlinked in many ways. In contrast to other software products for microarray analysis, the user is neither tied to a particular methodology nor needs to understand in detail the inner working of the algorithms. New researchers in the field can use the web-tool without the risk of deviating from standard validation guidelines.

For the next version of the server, we are planning to add a new module for co-expression network analysis and the possibility to integrate additional clinical or biological data.

## Availability and requirements

The web-application, video tutorials and an illustration of the features and workflow are freely accessible at .

## Authors' contributions

EG participated in the conceptual design of the web-application, implemented the algorithms and PHP-interface and drafted the manuscript. NK took part in the conceptual design of the web-application and helped to draft the manuscript. JMG helped to draft the manuscript. JMG and NK wrote the grant application upon which this project was built. All authors read, made corrections and approved the final manuscript.

## Supplementary Material

Additional file 1 **Results for an example analysis with ArrayMining.net**. The document provided contains the results and a discussion for an example analysis of microarray data with ArrayMining.net. Click here for file

Additional file 2 **Example VRML-visualization of an Independent Component Analysis**. This file contains an example VRML-visualization of an Independent Component Analysis for the microarray data set by Armstrong et al. [55] (a VRML browser plugin or viewing software is required to open the file). Click here for file
