# Supplementary material for: ArrayMining: a modular web-application for microarray analysis combining ensemble and consensus methods with cross-study normalization
Source: BMC Bioinformatics. 2009 Oct 28;10:358. doi: 10.1186/1471-2105-10-358 (PMC2776026; doi:10.1186/1471-2105-10-358)
Supplement: Additional file 1 — Results for an example analysis with ArrayMining.net. The document provided contains the results and a discussion for an example analysis of microarray data with ArrayMining.net. [file 1471-2105-10-358-S1.pdf]

ArrayMining: A modular web-application for microarray analysis combining ensemble and consensus methods with cross-study normalization - Supplementary Material

Enrico Glaab, Jonathan M. Garibaldi and Natalio Krasnogor

Table 1: **List of high scoring genes for the Armstrong et al. leukemia data set**

Affymetrix ID	Gene name	Gene symbol	F-statistic
32847_at	myosin, light polypeptide kinase	MYLK	159.59
1389_at	membrane metallo-endopeptidase (calla, cd10, neutral endopeptidase, enkephalinase)	MME	137.53
35164_at	wolfram syndrome 1 (wolframin)	WFS1	128
36239_at	pou domain, class 2, associating factor 1	POU2AF1	116.75
1325_at	smad, mothers against dpp homolog 1 (drosophila)	SMAD1	110.37
37280_at	smad, mothers against dpp homolog 1 (drosophila)	SMAD1	110.07
963_at	ligase iv, dna, atp-dependent	LIG4	89.77
34168_at	deoxynucleotidyltransferase, terminal	DNTT	89.31
40570_at	forkhead box o1a (rhabdomyosarcoma)	FOXO1	86.89
33412_at	lectin, galactoside-binding, soluble, 1 (galectin 1)	LGALS1	81.31
34950_at	zinc finger protein 423	ZNF423	78.12
37539_at	ral guanine nucleotide dissociation stimulator-like 1	RGL1	78
266_s_at	cd24 antigen (small cell lung carcinoma cluster 4 antigen)	CD24	76.74
31575_f_at	lectin, galactoside-binding, soluble, 1 (galectin 1)	LGALS1	76.04
37403_at	annexin a1	ANXA1	65.17
32838_at	myosin, heavy polypeptide 10, non-muscle	MYH10	64.83
31886_at	5'-nucleotidase, ecto (cd73)	NT5E	61.9
36021_at	lymphoid enhancer-binding factor 1	LEF1	61.51
1914_at	cyclin a1	CCNA1	60.3
34800_at	leucine-rich repeats and immunoglobulin-like domains 1	LRIG1	59.52
37680_at	a kinase (prka) anchor protein (gravin) 12	AKAP12	59.41
35648_at	autism susceptibility candidate 2	AUTS2	58.97
32533_s_at	vesicle-associated membrane protein 5 (myobrevin)	VAMP5	57.48
34990_at	set binding protein 1	SETBP1	56.39
38578_at	tumor necrosis factor receptor superfamily, member 7	CD27	54.38
1126_s_at	cd44 antigen (indian blood group)	CD44	53.39
41448_at	homeobox a10	HOXA10	48.08
36643_at	discoidin domain receptor family, member 1	DDR1	47.93
40282_s_at	complement factor d (adipsin)	CFD	47.77
35260_at	mlx interacting protein	MLXIP	42.92

List of the 30 top-ranked genes selected by the ENSEMBLE method on the leukemia data set by Armstrong et al. (all genes have a q-value below 0.002)

Example analysis of the leukemia microarray data set by Armstrong et al.

To illustrate the features available on ArrayMining.net, we have applied algorithms from different analysis modules to the well-known leukemia data set by Armstrong et al. In the following sections we will present the data set and pre-processing methods, discuss gene selection and clustering results obtained with our ensemble and consensus methods, as well as an example combination of the gene set analysis module with the class assignment module.

Data set and pre-processing

The leukemia data set by Armstrong et al. [1] contains expression values for 12,626 genes and 72 microarray samples, which are subdivided into three leukemia subtypes: Acute lymphoblastic leukemia (ALL, 24 samples), acute myelogenous leukemia (AML, 28 samples) and ALL with mixed-lineage leukemia gene translocation (MLL, 20 samples). Affymetrix U95A or U95A V2 oligonucleotide arrays [2] had been used in the study to obtain the experimental data.

To pre-process the raw data we applied the variance stabilizing normalization [3] using the expresso-package in the R statistical learning environment [4]. Moreover, we imposed thresholds based on the suggestions in the supplementary material of the original publication [1] and applied a fold-change filter to remove features with low variance (all gene vectors with less than a 5-fold change between the maximum and minimum expression value were discarded).

Gene selection results

In this section we present the results for a gene selection analysis on ArrayMining.net using the Armstrong et al. leukemia data and discuss the selected genes in detail. Table 1 shows the top 30 genes chosen by our ENSEMBLE method (the annotations in column 2 have been extracted from the DAVID [5] database; for two genes, SMAD1 and LGALS1, two different genetic probes matched to the same gene). We first identified those genes which were already identified as significantly differentially expressed in the original study on the Armstrong et al. data set [1] and then investigated the functional annotations of the genes based on the GO and KEGG data bases, ignoring very general annotations like “cell development” or “cell communication”. Finally, we searched for known functional associations between the genes and leukemia development in the biomedical literature.

As already stated in the study by Armstrong et al. many under-expressed genes in the MLL subtype have a function in early B-cell development. Among the genes belonging to this group, we identified *MME*, *CD24* and *DNTT*, *POU2AF1* and *LIG4* as significantly differentially expressed, which were already detected and discussed in the original study [1]. Similarly, our results confirm the finding by Armstrong et al. that certain adhesion molecules (*LGALS1*, *ANXA1*, *CD44*) are significantly over-expressed in MLL, as well as the myeloid-specific gene *CCNA1*. Other genes from our ranking which were already mentioned as distinguishing MLL from ALL by Armstrong et al. are *MYLK*, *FOXO1*, *MYH10* and *VAMP5* (see individual gene discussion below).

When investigating the available annotation data for the selected genes, we found that five genes are known to be involved in immune system processes (associated to the Gene Ontology terms “immune response” and “immune system process”). These are CD24, LIG4, CFD, POU2AF1 and CD27.

- *CD24* is a glycoprotein known to be involved in metastasis and highly expressed in many tumours, mediating apoptosis in precursor-B acute lymphoblastic leukemia cell lines [6].
- *LIG4* is the gene encoding the DNA Ligase IV protein, which joins double-strand breaks in the DNA, and a mutation in LIG4 has been suggested to confer a pre-disposition to leukemia [7].
- *CFD* (adipsin) is a serine protease involved in the alternative complement pathway as part of the

innate immune system. The gene is located in a chromosomal region which is known to be associated with myeloid cell differentiation by means of changes in chromatin organization [8].

- The gene *POU2AF1* (see Fig. 2) encodes a transcriptional regulator required by the immune system for the formation of germinal centers in lymph follicles after antigen contact and binding specifically to either the transcription factor OCT1 or OCT2 in the B-cell response to antigens [9, 10]. Deregulation of POU2AF1 by means of translocation has been implicated in lymphoma and leukemia development [11] and the gene's expression levels have previously been shown to vary across different lymphoma types by means of real-time quantitative PCR analysis [12].

- *CD27* is a tumor necrosis factor receptor which has been implicated in B-cell activation and found to be differentially expressed in normal B-cells and neoplastic B-cells [13].

Six genes in the ranking were found to be involved in apoptosis. Apart from the already discussed genes CD24, LIG4 and CD27 these include FOXO1, ANXA1 and LGALS1.

- *FOXO1* is a member of the forkhead family of transcription factors which has been associated with cell cycle arrest and apoptosis of hematopoietic cells [14].

- *ANXA1*, belonging to the adhesion molecules over-expressed in MLL (see above), is a phospholipid-binding protein with anti-inflammatory functions which might result from its inhibitory effect on the inflammation mediator phospholipase 2 [15]. It has been reported to be differentially expressed in various cancers and is used as marker in an assay to differentiate between hairy cell leukaemia and other B-cell malignant diseases [16, 17].

- *LGALS1* is another adhesion molecule over-expressed in MLL, which has been reported to induce apoptosis of human thymocytes [18] and interact with the oncogene H-Ras [19].

Three other genes from the ranking, ZNF423, LEF1 and VAMP5, are associated with the GO-term for cell differentiation.

- *ZNF423* is a transcription factor whose deregulated expression has been shown to contribute to the induction of the terminal phase of chronic myelogenous leukemia, known as "blast crisis", which is clinically similar to an acute leukemia [20].

- *LEF1*, Lymphoid enhancer-binding factor 1, is a transcription factor expressed in pre-B and T-cells. It has been implicated in leukemogenesis based on experiments in which mice, transplanted with bone marrow retrovirally transduced to express LEF1, developed B lymphoblastic and acute myeloid leukemia [21].

- *VAMP5* is a member of the family of vesicle-associated membrane proteins. Since the mRNA and protein levels of VAMP5 have been shown to be increased during in vitro myogenesis, it has been suggested that VAMP5 could be involved in vesicle trafficking events that are associated with myogenesis [22].

Among the top-ranking genes not discussed so far, four are involved in DNA-dependent regulation of transcription (GO-term 6355): HOXA10, SMAD1, MLXIP and SETBP1.

- *HOXA10* is transcription factor whose over-expression in murine hematopoietic cells has been reported to perturb myeloid and lymphoid differentiation leading to acute myeloid leukemia [23].

- *SMAD1* (see Fig. 2) is a transcriptional modulator and a component of the transforming growth factor (TGF)-beta signaling pathway, which plays a key role in cell differentiation and apoptosis pathways [24]. Different studies have revealed that mutations in SMAD-genes can cause a disruption of this pathway leading to various types of leukemia [25, 24].

- *MLXIP* is a protein interacting with MAX-like protein X (MLX), which plays a role in proliferation and differentiation. It has been shown that MAX and MAX-like proteins can form heterodimers with MAD family proteins which oppose the growth-promoting action of heterodimers between MAX and the oncogene MYC [26].
- *SETBP1* is a transcription factor involved in hematopoietic stem cell (HSC) regulation, and fusion of SETBP1 with another gene (NUP98) has been reported in T-cell lymphoblastic leukaemia [27].

Among the remaining genes in the ranking, which were not found in specific cancer-related GO or KEGG terms, we first discuss the six genes that were already identified by Armstrong et al. as discriminators between ALL and MLL: MME, CCNA1, DNTT, CD44, MYLK, MYH10.

- *MME* stands for the enzyme "membrane metallo- endopeptidase" and is also known as "common acute lymphoblastic leukemia antigen" (CALLA). The protein is involved in the degradation of secreted peptides and has been suggested to play a role at an early stage of lymphoid differentiation [28]. Furthermore, MME has been demonstrated to be expressed in most acute lymphoblastic lymphomas and in some B-cell and T-cell lymphomas [29].
- *CCNA1* is a cyclin protein involved in cell cycle regulation which is highly expressed in various myeloid leukemic cell lines and has therefore been implicated in germline meiotic cell cycle control [30].
- *DNTT* encodes a template-independent DNA polymerase which generates antigen receptor diversity by adding nucleotides at the junction of rearranged Ig heavy chainand T-cell receptor gene segments during the maturation of B- and T-cells [31, 32]. DNTT has been suggested as a marker distinguishing subtypes of lymphoid leukemias of childhood [33].
- *CD44* is a cell-surface protein with a great variety of functions resulting from a large number of splicing isoforms. It is involved in cell adhesion and migration processes and more specifically, lymphocyte activation, tumor metastasis and hematopoiesis [34]. The ligation of CD44 has been reported to reverse blockage of differentiation in human acute myeloid leukemia [35].
- *MYLK* (myosin light chain kinase, see Fig. 2) is an enzyme which phosphorylates myosin light chains to support the interaction of actin filaments with myosin and changed expression of MYLK leading to inhibition or potentiation of myosin II activation has been shown to delay or accelerate tumor necrosis factor-alpha (TNF)-induced apoptotic cell death [36]. Moreover, expression of MYLK is correlated with disease recurrence and distant metastasis in non-small cell lung cancer [37].
- *MYH10* (myosin heavy chain 10, non-muscle) is a gene coding for a myosin protein with putative functions in cytokinesis and cell shape, and has been reported to be down-regulated in patients with T-lineage acute lymphoblastic leukemia for whom induction therapy fails [38].

Finally, we discuss the genes which were neither found in cancer-specific GO or KEGG terms nor discussed as being functionally related to leukemia development in the paper by Armstong et al. These are WFS1, RGL1, NT5E, LRIG1, AKAP12, AUTS2 and DDR1.

- *WFS1* (see Fig. 2) has been investigated as a candidate glucocorticoid-response gene in childhood acute lymphoblastic leukemia (glucocorticoids mediate apoptosis of lymphoid cells and are therefore used in chemotherapy for lymphoid malignancies) [39].
- *RGL1* is a guanine nucleotide exchange factor and a proposed effector of the oncogene ras and other ras-like proteins [40].
- *NT5E* is an enzyme catalyzing the dephosphorylation of AMP and other nucleoside monophosphates and is used as a marker for lymphocyte differentiation [41]. The activity of NT5E has been observed to be strongly reduced in peripheral blood lymphocytes from B-Cell chronic lymphocytic leukemia patients in comparison to normal cells [42].

- *LRIG1* encodes a transmembrane protein which interacts with receptor tyrosine kinases of the epidermal growth factor receptor (EGFR) family and restricts growth factor signaling by enhancing receptor degradation [43]. The protein has been investigated as a potential tumor suppressor and was found to be down-regulated in conventional and papillary renal cell carcinomas [44].

- *AKAP12* is a tumor suppressor gene encoding a cell growth-related protein binding to the regulatory subunit of protein kinase A [45]. Based on real-time quantitative PCR measurements on 162 samples, AKAP12 expression has been found to be decreased in samples of acute leukaemia as compared to healthy controls and to be associated with an inferior overall survival [46].

- The function of *AUTS2* is currently unknown, but in patients with B-cell precursor acute lymphoblastic leukemia (BCP-ALL) fusions of AUTS2 with PAX5, an important regulator of B-cell development, have been reported [47].

- *DDR1* is a receptor tyrosine kinase which is up-regulated by p53 oncoprotein [48] and has been identified as significantly over-expressed in many human tumors [49, 50, 51].

In summary, almost all of the selected genes are either known oncogenes or tumor suppressor genes or have been suggested to be functionally associated with cancer progression or immune response processes. Differences in the expression status of these genes across different cancer types or different stages or subtypes of specific cancer diseases are therefore to be expected, which matches to the observation of differential expression across different leukemia subtypes on the Armstrong et al. data set. Although false positives cannot be ruled out in this approach, the functional annotations of the selected genes and the related findings from the literature suggest that this gene selection scheme is useful in prioritizing genes and proteins for investigating their potential involvement in explaining differences between diverse conditions of the biological system of interest.

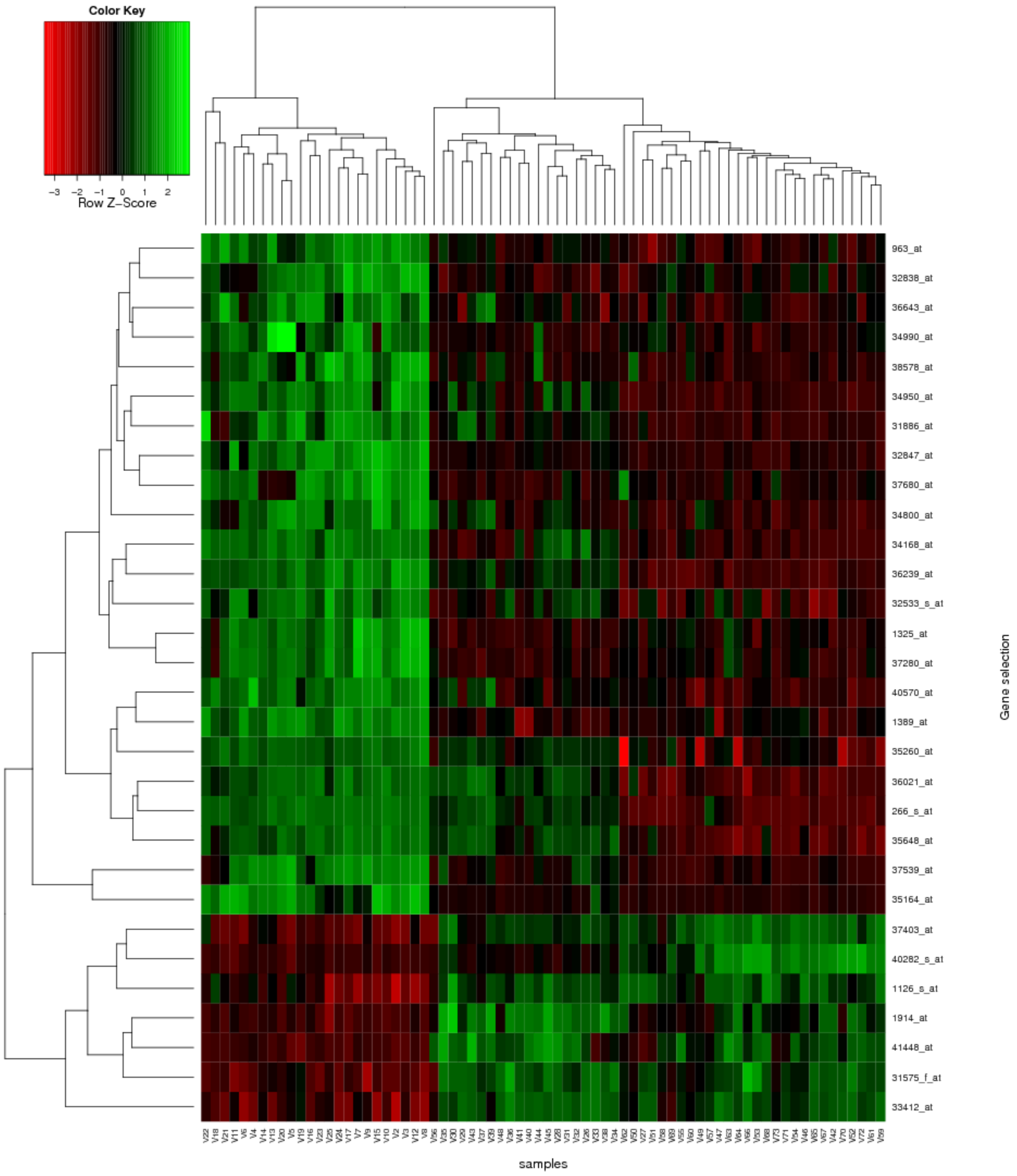

Figure 1: Heat map for the 30 genes selected by the ENSEMBLE method on the Armstrong et al. leukemia data set (rows correspond to genes, columns correspond to samples)

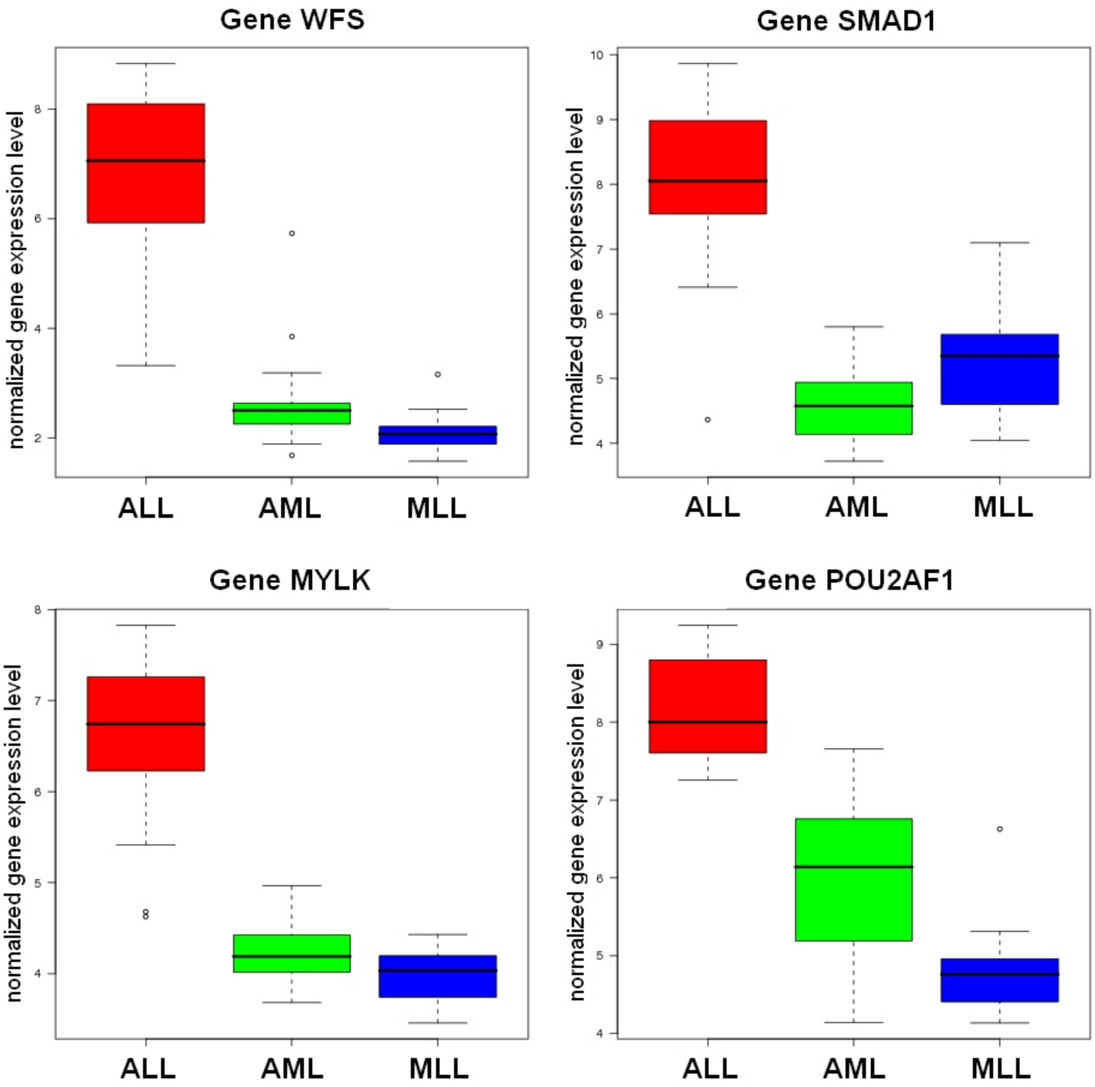

Figure 2: Four example box plots for genes selected by the ENSEMBLE method on the Armstrong et al. leukemia data set (the horizontal axis separates the tumor sample groups, the vertical axis corresponds to the normalized gene expression value)

Clustering results

As an example application of the class discovery module on ArrayMining.net, we present results obtained on the leukemia data set by Armstrong et al. using the ensemble clustering method. Prior to the analysis we standardized the samples by subtracting the median from the expression values and dividing by the median absolute deviation. Moreover, we applied a variance filter, retaining only the 2000 genes with the highest variance across the samples (both of these options are available on the class discovery interface on ArrayMining.net).

When comparing the estimated optimal number of clusters for different pairs of clustering methods and cluster validity indices, the great majority of approaches estimate 2 as the optimal number of clusters. In order to investigate this result in more detail, we manually inspected the optimal clustering results with regard to the validity indices for different algorithms and found that the methods could perfectly distinguish

Table 2: **Estimated number of clusters for the Armstrong et al. leukemia data set based on different clustering methods and validity indices**

Method	Validity index	Est. number of clusters
CLARA	C-index	2
SOTA	C-index	2
HYBRID	C-index	2
PAM	C-index	2
HIERARCHICAL	C-index	2
PAM	Calinski-Harabasz	2
CLARA	Calinski-Harabasz	2
HIERARCHICAL	Calinski-Harabasz	2
HYBRID	Calinski-Harabasz	2
KMEANS	Calinski-Harabasz	2
SOM	Calinski-Harabasz	2
PAM	Dunn	2
SOM	Dunn	2
HIERARCHICAL	Dunn	2
KMEANS	Dunn	2
SOTA	Dunn	2
DIANA	Dunn	2
KMEANS	Silhouette	2
SOM	Silhouette	2
SOTA	Silhouette	2
DIANA	Silhouette	2
CLARA	Silhouette	2
HIERARCHICAL	Silhouette	2
HYBRID	Silhouette	2
KMEANS	knn-Connectivity	2
SOM	knn-Connectivity	2
DIANA	knn-Connectivity	2
CLARA	knn-Connectivity	2
SOTA	knn-Connectivity	2
HYBRID	knn-Connectivity	2
HYBRID	Dunn	3
HIERARCHICAL	knn-Connectivity	3
SOM	C-index	4
DIANA	Calinski-Harabasz	4
PAM	Silhouette	5
CLARA	Dunn	6
KMEANS	C-index	7
DIANA	C-index	7
SOTA	Calinski-Harabasz	8
PAM	knn-Connectivity	8

List of the estimated number of clusters on the leukemia data set by Armstrong et al. based on pairwise combinations of 8 clustering methods and 5 validity indices

the Mixed Lineage Leukemia (MLL) subtype from the Acute Lymphoblastic Leukemia (ALL) subtype, but could not separate ALL from Acute Myeloid Leukemia (AML) and only partly separate MLL from AML samples. In most clusterings, all MLL samples were assigned to the same cluster and only some of the AML samples were assigned additionally to this cluster, but none of ALL samples.

Although the original grouping of samples into ALL, AML and MLL subtypes is based on objective criteria, several meaningful cluster structures might exist in the data and there is no single objective criterion as to what the “real” or “optimal” clustering structure is. However, we notice that MLL was assigned to a separate cluster from the ALL samples and only some of the AML samples were additionally assigned to

the MLL-cluster, which matches to the finding in the original study by Armstrong et al. according to which the MLL subtype is more similar to AML than to ALL (with regard to results from a principal component analysis and based on the expression of myeloid-specific genes in MLL).

The clustering results were also combined into a single representative clustering using our Simulated Annealing based consensus clustering method and the outcome was visualized using a principal component analysis plot (see Fig. 3). This plot confirms the observations from the manual inspection of the single algorithm clustering results according to which the MLL samples can perfectly be separated from the ALL group and are partly overlapping with the AML samples. A silhouette plots visualizes the silhouette widths for each sample as a reliability measure for the corresponding cluster assignment [52] (see Fig. 4).

When inspecting a 3-dimensional visualization of pre-filtered data using an Independent Component Analysis, a better separation of the tumor subtypes was observed, with no overlap between the ALL/MLL groups and the AML/ALL groups and only a small overlap between AML and MLL samples (see the VRML-file in the Supplementary Material). Moreover, density estimation contour surfaces revealed three regions of high data density (colored green in the VRML-file) corresponding to the three leukemia types.

On the whole, although only standard parameters had been used and all results were generated in an automatic process by the class discovery module, the clustering and visualization results enable the user to distinguish between major sample subgroups in the data (e.g. ALL vs. MLL) and provide other useful insights (e.g. MLL samples are more similar to AML samples than to ALL samples with regard to their expression profiles).

Gene set analysis results

To demonstrate the features of our gene set analysis module, we tested the enrichment of a collection of 37 cancer-related gene sets from the van Andel Institute in Michigan [53] in the leukemia data set by Armstrong et al. The module also provides access to Gene Ontology and KEGG gene sets, but these contain many very general gene sets (e.g. "cell cycle"-related genes) which are enriched in almost all microarray data sets and therefore only have a limited value for biological interpretation of the data.

We applied the MDS-GSA method using multi-dimensional scaling to combine the expression vectors for the genes in a gene set into a single signature vector (the "meta-gene"). To asses whether the enriched gene sets were differentially expressed across pairs of different sample groups the empirical Bayes t-statistic was used [54].

The q-value significance scores in the ranked table of gene sets, computed based on the Benjamini-Hochberg method, suggest that many cancer-related gene sets are significantly differentially expressed across the sample groups. We therefore only discuss the three gene sets with the smallest q-values as example cases: *VEGF down*, *ES M down* and *FH down*.

- The *VEGF down* gene set contains genes associated with vasculogenesis and angiogenesis obtained from a microarray study in which human umbilical cord vein endothelial cell (HUVEC) isolates were treated with vascular endothelial growth factor-A (VEGF-A) in low or high serum media. Apart from vasculogenesis and angiogenesis, VEGF has also been associated with growth, dissemination, metastasis and poor outcome in solid tumors and has been shown to be an independent predictor of outcome in patients with acute myeloid leukemia (AML) [55]. Moreover, an analysis of VEGF expression in the bone marrow of AML patients showed that VEGF is restricted to certain stages of differentiation and correlates with AML sub-categories [56]. The role of VEGF in myeloid leukemias is also highlighted by another study which showed that a broad spectrum of VEGF receptors is expressed in various myeloid neoplasms [57].

- The *ES M down* gene set was obtained from a study comparing the expression levels of genes in multipotent mesenchymal embryonic stem cells (ESM) against adult mesenchymal stem cells (MSC). It is composed of the genes which are significantly down-regulated in ESM cells as compared to MSCs. Similarities between stem cells and some cancer cells, e.g. the potential for self-renewal, have been widely discussed in the literature [58]. Another example for this is the cytokine "leukemia inhibitory

Table 3: **List of differentially expressed cancer-related gene sets for the Armstrong et al. leukemia data set**

Identifier	Pathway/function	PubMed/GEO ID	Q-values	F-score
VEGF down	Vasculogenesis and angiogenesis (Vascular endothelial growth factor dependent)	GEO: GDS495	1.60E-22	119.03
ES M down	Cell differentiation (differentially expressed in multipotent mesenchymal embryonic stem cells)	PMID: 15971941	3.00E-22	113.64
FH down	Differentially expressed in cells with fumarate-hydratase mutations	PMID: 16319128	3.90E-22	110.97
TNF 2 up	Inflammation response (Tumour necrosis factor regulated)	GEO: GSE2489	8.00E-22	107.03
E2F3 up	Cell cycle regulation/control of tumour suppressor genes (oncogene)	PMID: 16273092	6.40E-19	82.92
HYPER 2 down	Differential expression induced by hyperoxia	GEO: GSE489	8.00E-19	81.6
ES M up	Cell differentiation (differentially expressed in multipotent mesenchymal embryonic stem cells)	PMID: 15971941	9.50E-19	80.58
HYPER 2 up	Differential expression induced by hyperoxia	GEO: GSE489	1.30E-18	79.14
NFKB1 up	Inflammation response (Nuclear Factor-kB (NF-kB) dependent)	GEO: GSE2624	4.90E-18	74.83
HYPOXIA up	Differential expression induced by hypoxia (small set)	PMID: 16417408	6.40E-18	73.43
HGF 2 down	Proliferation and cell migration (Hepatocyte growth factor induced differential expression)	PMID: 16052207	6.40E-18	73.47
VEGF up	Vasculogenesis and angiogenesis (Vascular endothelial growth factor dependent)	GEO: GDS495	7.60E-18	72.66
HYPOXIA down	Differential expression induced by hypoxia	PMID: 16417408	2.70E-17	68.84
NFKB1 down	Inflammation response (Nuclear Factor-kB (NF-kB) dependent)	GEO: GSE2624	5.20E-17	66.76
HGF 2 up	Proliferation and cell migration (Hepatocyte growth factor induced differential expression)	PMID: 16052207	8.00E-16	59.3
MET up	Met-regulated expression signature	PMID: 16710476	3.10E-14	50.24
HRAS down	Cell division regulation and growth factor stimulation (oncogene)	PMID: 16273092	3.50E-14	49.77
WND up	Wound healing processes	PMID: 14737219	6.20E-14	48.35
SRC down	Family of proto-oncogenic tyrosine kinases	PMID: 16273092	7.50E-14	47.8
CMYC down	Gene regulation (oncogene)	PMID: 16273092	1.60E-13	46.04
NFKB1 2 down	Inflammation response (Nuclear Factor-kB (NF-kB) dependent)	GEO: GSE2489	3.20E-13	44.35
HYPER up	Differential expression induced by hyperoxia	GEO: GSE489	7.60E-13	42.4
WND down	Wound healing processes	PMID: 14737219	5.90E-12	38.08
FH up	Differentially expressed in cells with fumarate-hydratase mutations	PMID: 16319128	1.10E-11	36.68
TNF 2 down	Inflammation response (Tumour necrosis factor regulated)	GEO: GSE2489	2.60E-11	35.04
SRC up	Family of proto-oncogenic tyrosine kinases	PMID: 16273092	3.10E-11	34.6
HRAS up	Cell division regulation, growth factor stimulation (oncogene)	PMID: 16273092	4.20E-11	33.93
BRAF down	Cell-signaling and cell growth (oncogene)	PMID: 15048078	6.80E-10	28.72
TFA change	Chromosomal instability signature (Total function aneuploidy)	PMID: 16921376	4.30E-08	21.77
TNF up	Inflammation response (Tumour necrosis factor regulated)	GEO: GSE2624	9.80E-08	20.44
BRAF up	Cell-signaling and cell growth (oncogene)	PMID: 15048078	2.00E-06	15.9
E2F3 down	control of tumour suppressor genes (oncogene)	PMID: 16273092	4.30E-06	14.8
NFKB1 2 up	Inflammation response (Nuclear Factor-kB (NF-kB) dependent)	GEO: GSE2489	4.60E-06	14.67
HYPOXIA up	Differential expression induced by hypoxia (large set)	PMID: 16417408	3.60E-05	11.86
CMYC up	Gene regulation (oncogene)	PMID: 16273092	7.80E-05	10.81
TNF down	Inflammation response (Tumour necrosis factor regulated)	GEO: GSE2624	1.60E-04	9.89
HYPER down	Differential expression induced by hyperoxia	GEO: GSE489	3.00E-02	3.67

List of cancer-related gene sets from the van Andel institute in Michigan which were identified as differentially expressed on the Armstrong et al. leukemia data (using the GSA-MDS method)

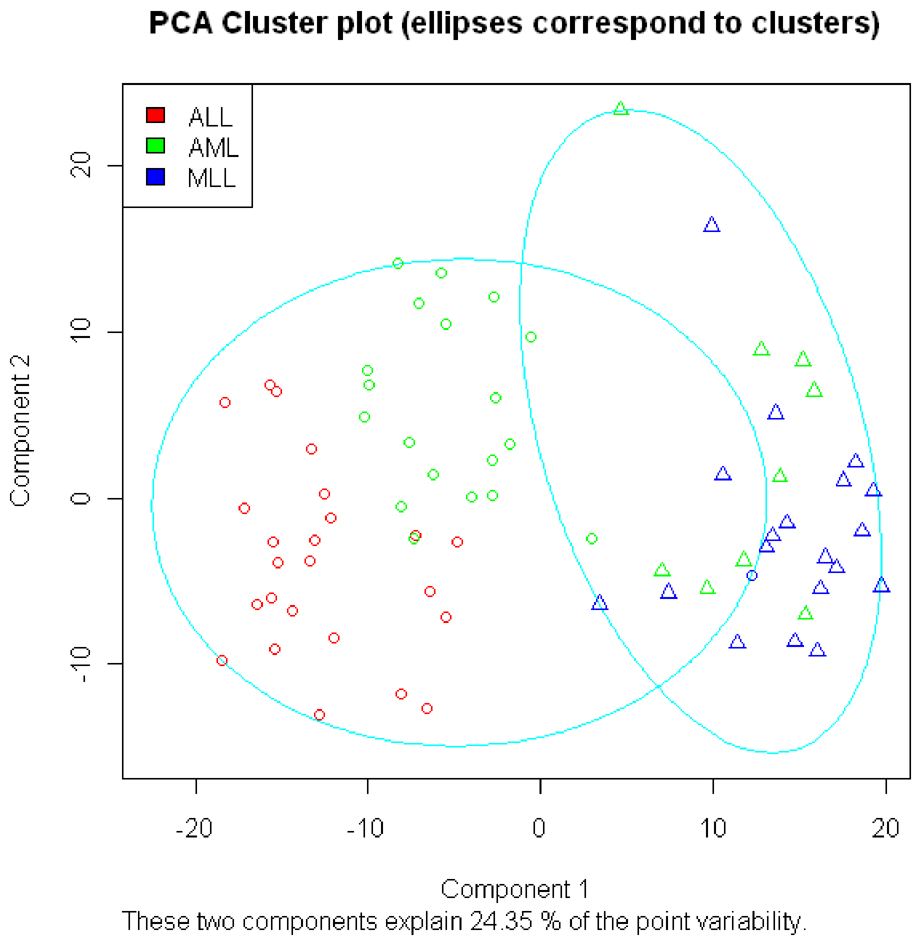

Figure 3: Principal Component Analysis based visualization of clustering results for the Armstrong et al. leukemia data set - the two clusters identified by the consensus clustering are shown as ellipses and the corresponding samples are encoded by different symbols (triangles and circles); the tumor types of the samples are represented by different colors (ALL = red, AML = green and MLL = blue)

factor" (LIF), which induces terminal differentiation of myeloid leukemia cells and also plays an important role in the regulation of signaling in embryonic stem cells [59].

- The *FH down* gene set is derived from microarray experiments studying differences in gene expression patterns in uterine fibroids caused by mutations in the fumarate hydratase (FH) gene. It contains genes which were significantly down-regulated in fibroids containing FH-mutations as opposed to fibroids with wild-type FH. Mutations and other defects in mitochondrial enzymes like FH have been reported to predispose to tumorigenesis. Mitochondrial DNA alterations have for example been implicated in the transformation of myelodysplastic syndromes into acute leukemia [60].

In addition to the ranking of gene sets, a heat map was generated to visualize the meta-gene expression values across different samples and sample groups (see Fig. 5). Although the interpretation of the meta-gene expression vectors, obtained from combining multiple genes into a single vector by means of multi-dimensional scaling, is not as straightforward as in the case of single gene expression vectors, the heat map suggests that the derived meta-genes can to certain extent distinguish between the three leukemia classes. Moreover, in agreement with our previous findings in the clustering analysis, the heat map indicates that the AML samples are more similar to the MLL than to the ALL samples. A subgroup of about 8 samples

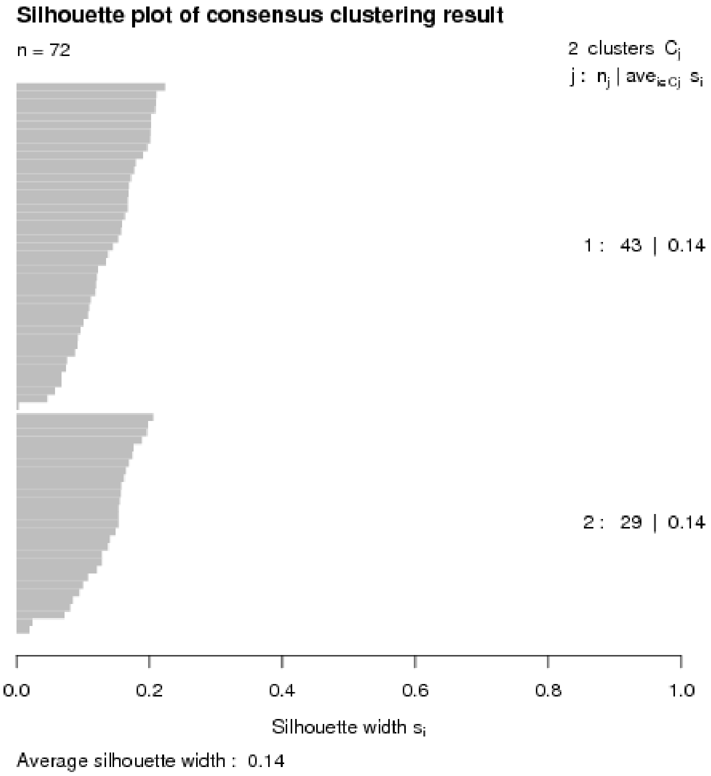

Figure 4: Silhouette plot visualizing the Silhouette width for each sample by horizontal bars as a confidence measure for the sample's cluster assignment (based on the consensus clustering results for the Armstrong et al. leukemia data set)

in the AML-group (the columns on the right in the heat map) appears to bear a particularly close similarity to the MLL group with regard to the meta-gene expression profile.

These preliminary gene set analysis results suggest that this type of analysis can provide additional insights as compared to a so-called "singular enrichment analysis", in which the genes are first pre-selected using feature selection before testing the enrichment of certain functional gene groups (see gene selection results above). Moreover, by using cancer-related gene sets instead of Gene Ontology or KEGG derived gene sets, the annotations of the enriched gene sets are likely to be more specific and more informative for the biological interpretation of the data.

Prediction results

Using the class assignment module on ArrayMining.net we illustrate how sample classification results can be obtained in an automatic fashion by uploading data on this module and how the results can be improved by using features obtained from another analysis module (in this case, the gene set analysis module).

We applied a 10-fold external cross-validation [61] analysis on the leukemia data by Armstrong et al. using our ENSEMBLE prediction method and the empirical Bayes t-statistic for feature selection [54]. Moreover, we applied the same cross-validation scheme and the same predictor to meta-gene expression values derived from a gene set analysis on the same data set using the GSA-MDS method and Gene Ontology derived gene sets (we chose the Gene Ontology data base here, because it contains a much larger number of gene sets than our collection of cancer-related gene sets).

Based on single genes as features an average cross-validated accuracy of 80.9% (±14%) was obtained,

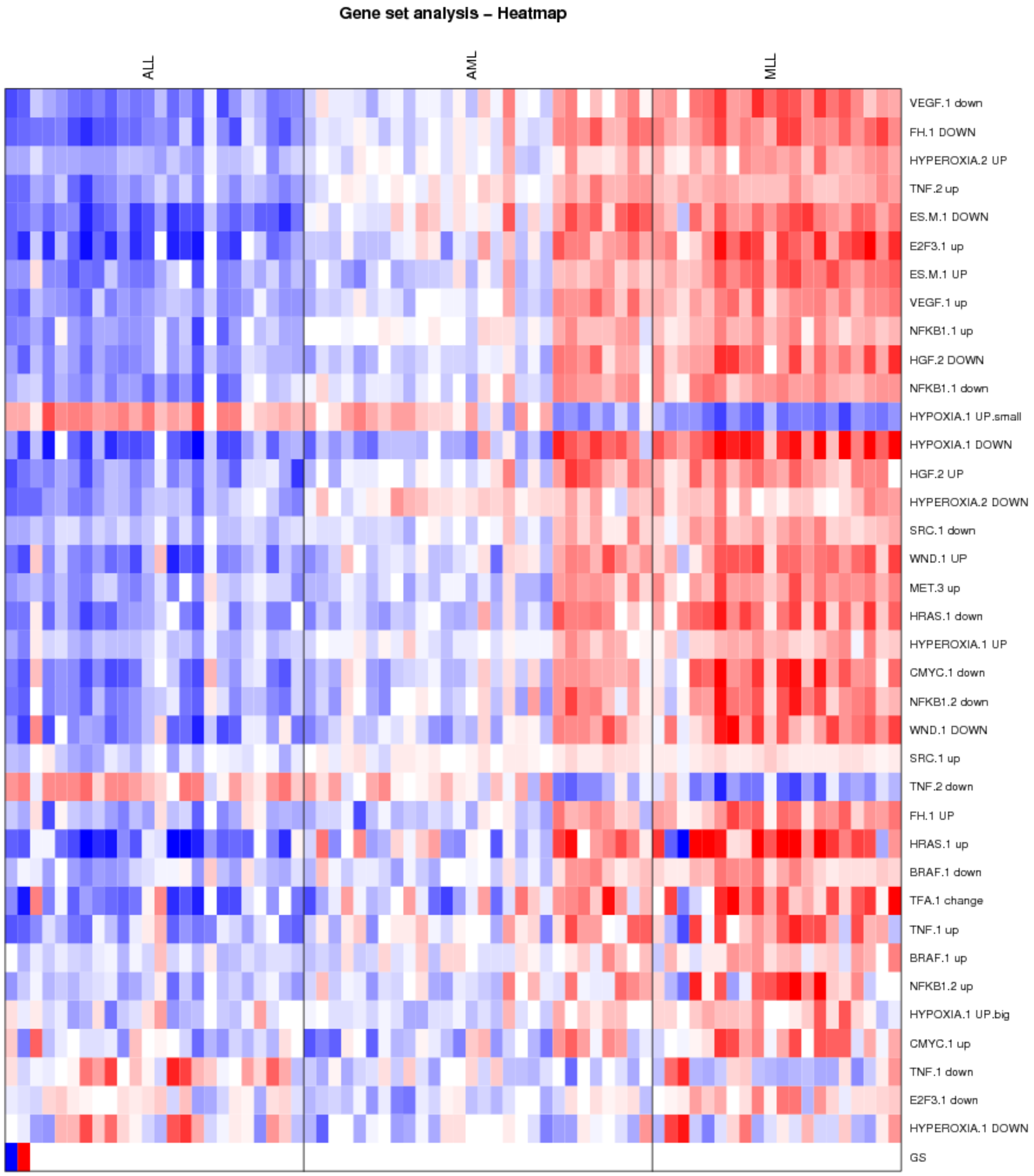

Figure 5: Heat map showing differentially expressed gene sets for the Armstrong et al. leukemia data set (rows correspond to gene sets, columns correspond to samples)

whereas 87.5% (±11%) accuracy were reached when using the meta-genes as input. Although some studies have reported higher accuracies on this data set, we think these are reasonable results for a 3-class microarray classification problem with 72 samples, using an almost fully automated process and an external cross-validation scheme [61].

Since the classification results are affected by high variance, which is common in microarray studies with small sample sizes, we additionally report results for three other gene expression cancer data sets using the same methodology as above (see Tab. 4). Similar trends can be observed across the different data sets: Using meta-genes representing biological pathways as features similar accuracies and standard deviations were reached as based on single genes. This suggests that the user can generate models of similar predictive quality based on biological pathways and on single genes, enabling two different model-based interpretations of the data.

In summary, these example results suggest that the class assignment module can be a helpful tool to compare cross-validated prediction accuracies for different data sets and methods. In combination with the gene set analysis module, prediction models based on biological pathways as features with high classification accuracies can be obtained based on an almost fully automated process.

Table 4: **Comparison of sample classification results for different data sets**

	Predictors:			
	Single genes		Gene sets ("Meta-genes")	
Data sets:	accuracy (%)	stddev.	accuracy (%)	stddev.
Leukemia [1]	80.9	14	87.5	11
Prostate cancer [62]	92.3	9	89.5	10
DLBCL [63]	95.0	9	95.0	6
T-Cell Lymphoma [64]	81.0	13	82.6	14

Comparison of 10-fold cross-validation sample classification results obtained on different microarray cancer data sets using both single genes and summarized gene sets as features

References

[1] Armstrong S, Staunton J, Silverman L, Pieters R, den Boer M, Minden M, Sallan S, Lander E, Golub T, Korsmeyer S: **MLL translocations specify a distinct gene expression profile that distinguishes a unique leukemia**. *Nat Genet* 2001, **30**:41–47.

[2] Affymetrix: *Affymetrix Microarray Suite User Guide, Version 5*. Affymetrix, Santa Clara, CA 2001.

[3] Huber W, von Heydebreck A, Sültmann H, Poustka A, Vingron M: **Variance stabilization applied to microarray data calibration and to the quantification of differential expression**. *Bioinformatics* 2002, **18**:96–104.

[4] Ihaka R, Gentleman R: **R: A Language for Data Analysis and Graphics**. *J Comput Graph Stat* 1996, **5**(3):299–314.

[5] Dennis Jr G, Sherman B, Hosack D, Yang J, Gao W, Lane H, Lempicki R: **DAVID: Database for Annotation, Visualization, and Integrated Discovery**. *Genome Biol* 2003, **4**(9):R60.

[6] Taguchi T, Kiyokawa N, Mimori K, Suzuki T, Sekino T, Nakajima H, Saito M, Katagiri Y, Matsuo N, Matsuo Y, et al.: **Pre-B Cell Antigen Receptor-Mediated Signal Inhibits CD24-Induced Apoptosis in Human Pre-B Cells 1**. *J Immunol* 2003, **170**:252–260.

[7] Riballo E, Critchlow S, Teo S, Doherty A, Priestley A, Broughton B, Kysela B, Beamish H, Plowman N, Arlett C, et al.: **Identification of a defect in DNA ligase IV in a radiosensitive leukaemia patient**. *Curr Biol* 1999, **9**:699–702.

[8] Wong E, Jenne D, Zimmer M, Porter S, Gilks C: **Changes in chromatin organization at the neutrophil elastase locus associated with myeloid cell differentiation**. *Blood* 1999, **94**(11):3730.

[9] Hanamura I, Iida S, Ueda R, Kuehl M, Cullraro C, Bergsagel L, Sawyer J, Barlogie B, Shaughnessy J: **Identification of Three Novel Chromosomal Translocation Partners Involving the Immunoglobulin Loci in Newly Diagnosed Myeloma and Human Myeloma Cell Lines.** *Blood* 2005, **106**(11):1552–1552.

[10] Andersen B, Rosenfeld M: **POU Domain Factors in the Neuroendocrine System: Lessons from Developmental Biology Provide Insights into Human Disease 1**. *Endocr Rev* 2001, **22**:2–35.

[11] Galiéque Z, Quief S, Hildebrand M, Denis C, Lecocq G, Collyn-d'Hooghe M, Bastard C, Yuille M, Dyer M, Kerckaert J: **The B cell transcriptional coactivator BOB1/OBF1 gene fuses to the LAZ3/BCL6 gene by t(3;11)(q27;q23.1) chromosomal translocation in a B cell leukemia line (Karpas 231)**. *Leukemia* 1996, **10**(4):579.

[12] Shin M, Fredrickson T, Hartley J, Suzuki T, Agaki K, Morse H: **High-throughput retroviral tagging for identification of genes involved in initiation and progression of mouse splenic marginal zone lymphomas**. *Cancer Res* 2004, **64**(13):4419–4427.

[13] Lens S, De Jong R, Hintzen R, Koopman G, Van Lier R, Van Oers R: **CD27–CD70 interaction: unravelling its implication in normal and neoplastic B-cell growth**. *Leuk Lymphoma* 1995, **18**:51–59.

[14] Jagani Z, Singh A, Khosravi-Far R: **FoxO tumor suppressors and BCR–ABL-induced leukemia: A matter of evasion of apoptosis**. *BBA-Reviews on Cancer* 2008, **1785**:63–84.

[15] Alldridge L, Bryant C: **Annexin 1 regulates cell proliferation by disruption of cell morphology and inhibition of cyclin D1 expression through sustained activation of the ERK1/2 MAPK signal**. *Exp Cell Res* 2003, **290**:93–107.

[16] Silistino-Souza R, Rodrigues-Lisoni F, Cury P, Maniglia J, Raposo L, Tajara E, Christian H, Oliani S: **Annexin 1: differential expression in tumor and mast cells in human larynx cancer**. *Int J Cancer* 2007, **120**(12).

[17] Falini B, Tiacci E, Liso A, Basso K, Sabattini E, Pacini R, Foa R, Pulsoni A, Favera R, Pileri S: **Simple diagnostic assay for hairy cell leukaemia by immunocytochemical detection of annexin A1 (ANXA1)**. *Lancet* 2004, **363**(9424):1869–1870.

[18] Perillo N, Uittenbogaart C, Nguyen J, Baum L: **Galectin-1, an endogenous lectin produced by thymic epithelial cells, induces apoptosis of human thymocytes**. *J Exp Med* 1997, **185**(10):1851–1858.

[19] Paz A, Haklai R, Elad-Sfadia G, Ballan E, Kloog Y: **Galectin-1 binds oncogenic H-Ras to mediate Ras membrane anchorage and cell transformation.** *Oncogene* 2001, **20**(51):7486.

[20] Miyazaki K, Yamasaki N, Oda H, Kuwata T, Kanno Y, Miyazaki M, Komeno Y, Kitaura J, Honda Z, Warming S, et al.: **Enhanced expression of p210BCR/ABL and aberrant expression of Zfp423/ZNF423 induce blast crisis of chronic myelogenous leukemia**. *Blood* 2009, **113**(19):4702.

[21] Petropoulos K, Arseni N, Schessl C, Stadler C, Rawat V, Deshpande A, Heilmeier B, Hiddemann W, Quintanilla-Martinez L, Bohlander S, et al.: **A novel role for Lef-1, a central transcription mediator of Wnt signaling, in leukemogenesis**. *J Exp Med* 2008, **205**(3):515.

[22] Pfeffer S, Zeng Q, Subramaniam V, Wong S, Tang B, Parton R, Rea S, James D, Hong W: **A novel synaptobrevin/VAMP homologous protein (VAMP5) is increased during in vitro myogenesis and present in the plasma membrane**. *Mol Biol Cell* 1998, **9**(9):2423–2437.

[23] Thorsteinsdottir U, Sauvageau G, Hough M, Dragowska W, Lansdorp P, Lawrence H, Largman C, Humphries R: **Overexpression of HOXA10 in murine hematopoietic cells perturbs both myeloid and lymphoid differentiation and leads to acute myeloid leukemia**. *Mol Cell Biol* 1997, **17**:495–505.

[24] Downing J: **TGF-β signaling, tumor suppression, and acute lymphoblastic leukemia**. *N Engl J Med* 2004, **351**(6):528–530.

[25] Imai Y, Kurokawa M, Izutsu K, Hangaishi A, Maki K, Ogawa S, Chiba S, Mitani K, Hirai H: **Mutations of the Smad 4 gene in acute myelogeneous leukemia and their functional implications in leukemogenesis**. *Oncogene* 2001, **20**:88–96.

[26] Billin A, Eilers A, Queva C, Ayer D: **Mlx, a novel Max-like BHLHZip protein that interacts with the Max network of transcription factors**. *J Biol Chem* 1999, **274**(51):36344–36350.

[27] Panagopoulos I, Kerndrup G, Carlsen N, Strombeck B, Isaksson M, Johansson B: **Fusion of NUP98 and the SET binding protein 1 (SETBP1) gene in a paediatric acute T cell lymphoblastic leukaemia with t (11; 18)(p15; q12).** *Br J Haematol* 2007, **136**(2):294.

[28] Shipp M, Vijayaraghavan J, Schmidt E, Masteller E, D'Adamio L, Hersh L, Reinherz E: **Common Acute Lymphoblastic Leukemia Antigen (CALLA) is Active Neutral Endopeptidase 24.11 ("Enkephalinase"): Direct Evidence by cDNA Transfection Analysis**. *Proc Natl Acad Sci U S A* 1989, **86**:297–301.

[29] Ritz J, Nadler L, Bhan A, Notis-McConarty J, Pesando J, Schlossman S: **Expression of common acute lymphoblastic leukemia antigen (CALLA) by lymphomas of B-cell and T-cell lineage**. *Blood* 1981, **58**(3):648–652.

[30] Yang R, Morosetti R, Koeffler H: **Characterization of a second human cyclin A that is highly expressed in testis and in several leukemic cell lines**. *Cancer Res* 1997, **57**(5):913–920.

[31] Komori T, Okada A, Stewart V, Alt F: **Lack of N regions in antigen receptor variable region genes of TdT-deficient lymphocytes**. *Science* 1993, **261**(5125):1171–1175.

[32] **GeneCards database entry: DNTT** [`http://www.genecards.org/cgi-bin/carddisp.pl?gene=Dntt`].

[33] Coleman M, Greenwood M, Hutton J, Holland P, Lampkin B, Krill C, Kastelic J: **Adenosine deaminase, terminal deoxynucleotidyl transferase (TdT), and cell surface markers in childhood acute leukemia**. *Blood* 1978, **52**(6):1125–1131.

[34] Guenthert U: **CD44: a multitude of isoforms with diverse functions.** *Curr Top Microbiol Immunol* 1993, **184**:47.

[35] Charrad R, Li Y, Delpech B, Balitrand N, Clay D, Jasmin C, Chomienne C, Smadja-Joffe F: **Ligation of the CD44 adhesion molecule reverses blockage of differentiation in human acute myeloid leukemia**. *Nat Med* 1999, **5**(6):669–676.

[36] Jin Y, Atkinson S, Marrs J, Gallagher P: **Myosin II light chain phosphorylation regulates membrane localization and apoptotic signaling of tumor necrosis factor receptor-1**. *J Biol Chem* 2001, **276**(32):30342–30349.

[37] Minamiya Y, Nakagawa T, Saito H, Matsuzaki I, Taguchi K, Ito M, Ogawa J: **Increased expression of myosin light chain kinase mRNA is related to metastasis in non-small cell lung cancer**. *Tumor Biol* 2005, **26**(3):153–157.

[38] Winter S, Jiang Z, Khawaja H, Griffin T, Devidas M, Asselin B, Larson R: **Identification of genomic classifiers that distinguish induction failure in T-lineage acute lymphoblastic leukemia: a report from the Children's Oncology Group**. *Blood* 2007, **110**(5):1429.

[39] Schmidt S, Rainer J, Riml S, Ploner C, Jesacher S, Achmuller C, Presul E, Skvortsov S, Crazzolara R, Fiegl M, et al.: **Identification of glucocorticoid-response genes in children with acute lymphoblastic leukemia**. *Blood* 2006, **107**(5):2061–2069.

[40] Kiel C, Foglierini M, Kuemmerer N, Beltrao P, Serrano L: **A genome-wide Ras-effector interaction network**. *J Mol Biol* 2007, **370**(5):1020–1032.

[41] Resta R, Yamashita Y, Thompson L: **Ecto-enzyme and signaling functions of lymphocyte CD73.** *Immunol Rev* 1998, **161**:95.

[42] Rosi F, Carlucci F, Marinello E, Tabucchi A: **Ecto-5-nucleotidase in B-cell chronic lymphocytic leukemia**. *Biomed Pharmacother* 2002, **56**(2):100–104.

[43] Gur G, Rubin C, Katz M, Amit I, Citri A, Nilsson J, Amariglio N, Henriksson R, Rechavi G, Hedman H, et al.: **LRIG1 restricts growth factor signaling by enhancing receptor ubiquitylation and degradation**. *EMBO J* 2004, **23**(16):3270.

[44] Thomasson M, Hedman H, Guo D, Ljungberg B, Henriksson R: **LRIG1 and epidermal growth factor receptor in renal cell carcinoma: a quantitative RT–PCR and immunohistochemical analysis**. *Br J Cancer* 2003, **89**(7):1285–1289.

[45] Choi M, Jong H, Kim T, Song S, Lee D, Lee J, Kim T, Kim N, Bang Y: **AKAP12/Gravin is inactivated by epigenetic mechanism in human gastric carcinoma and shows growth suppressor activity**. *Oncogene* 2004, **23**(42):7095–7103.

[46] Yildirim M, Paydas S, Tanriverdi K: **Gravin gene expression in acute leukaemias: Clinical importance and review of the literature**. *Leuk Lymphoma* 2007, **48**(6):1167–1172.

[47] Nebral K: **Detection and characterization of PAX5 aberrations in childhood acute lymphoblastic leukemia**. *PhD thesis*, University of Vienna, Life Sciences Department 2008.

[48] Sakuma S, Saya H, Tada M, Nakao M, Fujiwara T, Roth J, Sawamura Y, Shinohe Y, Abe H: **Receptor protein tyrosine kinase DDR is up-regulated by p53 protein**. *FEBS Lett* 1996, **398**(2-3):165–169.

[49] Johnson J, Edman J, Rutter W: **A receptor tyrosine kinase found in breast carcinoma cells has an extracellular discoidin I-like domain**. *Proc Natl Acad Sci U S A* 1993, **90**(12):5677–5681.

[50] Nemoto T, Ohashi K, Akashi T, Johnson J, Hirokawa K: **Overexpression of protein tyrosine kinases in human esophageal cancer**. *Pathobiology* 1997, **65**:195–203.

[51] Weiner H, Huang H, Zagzag D, Boyce H, Lichtenbaum R, Ziff E: **Consistent and selective expression of the discoidin domain receptor-1 tyrosine kinase in human brain tumors**. *Neurosurgery* 2000, **47**(6):1400.

[52] Rousseeuw P: **Silhouettes: a graphical aid to the interpretation and validation of cluster analysis**. *J Comput Appl Math* 1987, **20**:53–65.

[53] Kim S, Volsky D: **PAGE: parametric analysis of gene set enrichment**. *BMC Bioinformatics* 2005, **6**:144.

[54] Walker W, Liao I, Gilbert D, Wong B, Pollard K, McCulloch C, Lit L, Sharp F: **Empirical Bayes accomodation of batch-effects in microarray data using identical replicate reference samples: application to RNA expression profiling of blood from Duchenne muscular dystrophy patients**. *BMC Genomics* 2008, **9**:494.

[55] Aguayo A, Estey E, Kantarjian H, Mansouri T, Gidel C, Keating M, Giles F, Estrov Z, Barlogie B, Albitar M: **Cellular vascular endothelial growth factor is a predictor of outcome in patients with acute myeloid leukemia**. *Blood* 1999, **94**(11):3717.

[56] Ghannadan M, Wimazal F, Simonitsch I, Sperr W, Mayerhofer M, Sillaber C, Hauswirth A, Gadner H, Chott A, Horny H, et al.: **Immunohistochemical detection of VEGF in the bone marrow of patients with acute myeloid leukemia**. *Am J Clin Pathol* 2003, **119**(5):663–671.

[57] Vales A, Kondo R, Aichberger K, Mayerhofer M, Kainz B, Sperr W, Sillaber C, J
"ager U, Valent P: **Myeloid leukemias express a broad spectrum of VEGF receptors including neuropilin-1 (NRP-1) and NRP-2.** *Leuk Lymphoma* 2007, **48**(10):1997.

[58] Reya T, Morrison S, Clarke M, Weissman I: **Stem cells, cancer, and cancer stem cells**. *Nature* 2001, **414**(6859):105–111.

[59] Boeuf H, Hauss C, Graeve F, Baran N, Kedinger C: **Leukemia inhibitory factor-dependent transcriptional activation in embryonic stem cells**. *J Cell Biol* 1997, **138**(6):1207–1217.

[60] Linnartz B, Anglmayer R, Zanssen S: **Comprehensive scanning of somatic mitochondrial DNA alterations in acute leukemia developing from myelodysplastic syndromes**. *Cancer Res* 2004, **64**(6):1966–1971.

[61] Wood I, Visscher P, Mengersen K: **Classification based upon gene expression data: bias and precision of error rates**. *Bioinformatics* 2007, **23**(11):1363.

[62] Singh D, Febbo P, Ross K, Jackson D, Manola J, Ladd C, Tamayo P, Renshaw A, D'Amico A, Richie J, et al.: **Gene expression correlates of clinical prostate cancer behavior**. *Cancer Cell* 2002, **1**(2):203–209.

[63] Shipp M, Ross K, Tamayo P, Weng A, Kutok J, Aguiar R, Gaasenbeek M, Angelo M, Reich M, Pinkus G, et al.: **Diffuse large B-cell lymphoma outcome prediction by gene-expression profiling and supervised machine learning**. *Nat Med* 2002, **8**:68–74.

[64] Shin J, Monti S, Aires D, Duvic M, Golub T, Jones D, Kupper T: **Lesional gene expression profiling in cutaneous T-cell lymphoma reveals natural clusters associated with disease outcome**. *Blood* 2007, **110**(8):3015.
